# Objectively Measured Physical Activity and Fat Mass in a Large Cohort of Children 

**DOI:** 10.1371/journal.pmed.0040097

**Published:** 2007-03-20

**Authors:** Andy R Ness, Sam D Leary, Calum Mattocks, Steven N Blair, John J Reilly, Jonathan Wells, Sue Ingle, Kate Tilling, George Davey Smith, Chris Riddoch

**Affiliations:** 1 Department of Oral and Dental Science, University of Bristol, United Kingdom; 2 Department of Social Medicine, University of Bristol, Bristol, United Kingdom; 3 Arnold School of Public Health, University of South Carolina, Columbia, South Carolina, United States of America; 4 Division of Developmental Medicine, University of Glasgow, Glasgow, United Kingdom; 5 Medical Research Council Childhood Nutrition Research Centre, Institute of Child Health, University College London, London, United Kingdom; 6 London Sport Institute, Middlesex University, London, United Kingdom; London School of Hygiene and Tropical Medicine, United Kingdom

## Abstract

**Background:**

Previous studies have been unable to characterise the association between physical activity and obesity, possibly because most relied on inaccurate measures of physical activity and obesity.

**Methods and Findings:**

We carried out a cross sectional analysis on 5,500 12-year-old children enrolled in the Avon Longitudinal Study of Parents and Children. Total physical activity and minutes of moderate and vigorous physical activity (MVPA) were measured using the Actigraph accelerometer. Fat mass and obesity (defined as the top decile of fat mass) were measured using the Lunar Prodigy dual x-ray emission absorptiometry scanner. We found strong negative associations between MVPA and fat mass that were unaltered after adjustment for total physical activity. We found a strong negative dose-response association between MVPA and obesity. The odds ratio for obesity in adjusted models between top and the bottom quintiles of minutes of MVPA was 0.03 (95% confidence interval [CI] 0.01–0.13, *p*-value for trend <0.0001) in boys and 0.36 (95% CI 0.17–0.74, *p*-value for trend = 0.006) in girls.

**Conclusions:**

We demonstrated a strong graded inverse association between physical activity and obesity that was stronger in boys. Our data suggest that higher intensity physical activity may be more important than total activity.

## Abstract 2

A study with 5,500 12-year-olds shows an inverse association between physical activity and obesity, which is stronger in boys, and suggests that high-intensity activity is more important than total activity.

## Editors' Summary

**Background.:**

Obesity is a serious risk factor for many health problems, including heart attack, stroke, diabetes, cancers, and arthritis. The condition has become much more common over the last few decades, particularly in developed countries. The reasons for this increase are not fully understood; many factors—including changes in diet, lifestyle, and society as a whole—have been implicated. However, it is accepted that individual people who have become obese have been in a long-term state of “energy imbalance”—i.e., they have consumed more energy (usually measured in kilocalories, also termed Calories) than they have used up in physical activity. There is a continuing debate as to which is most important—eating too much food or the lack of sufficient activity. However, in several countries it looks like, on average, people are eating less than they were a few years ago and this suggests that a decline in physical activity could be the key factor in many cases of obesity.

**Why Was This Study Done?:**

It is actually quite hard to measure how much physical activity someone is performing and how many Calories they are using up in the process. Thus it has not been possible to prove that obese people do less physical activity than people of normal weight. It is also hard to define obesity! The usual method involves making a calculation based on height and weight but this is often criticized, as a person of above-average weight may be “carrying” a lot of fat or a lot of muscle. The researchers wanted to use new, accurate techniques to record physical activity and to measure fat mass, in order to see whether there is a difference between people who are obese and other people in terms of their activity level.

**What Did the Researchers Do and Find?:**

They did their work within a very large UK project called “the Avon Longitudinal Study of Parents and Children,” which is looking at many aspects of health. They did their research on 5,500 children who are a part of this study. They measured total physical activity and minutes of moderate and vigorous physical activity using a device called an Actigraph accelerometer. Fat mass was measured using a Lunar Prodigy dual x-ray emission absorptiometry scanner. The top 10% of the children, in terms of fat mass, were considered to be obese. Analysis of the results showed a consistent trend—the greater the fat mass the lower the level of physical activity. This effect (or “association”) was greater in boys than in girls; in other words, when the results were put on a graph the slope of the graph was steepest for the boys. It was also noted that the association between physical activity and obesity appeared to be due to moderate and vigorous physical activity rather than all physical activity.

**What Do These Findings Mean?:**

The researchers note that their study does have limitations. In particular, they discuss the so-called “direction of causality”—in other words it is possible that, instead of becoming obese because of a lack of activity, obese children may be restricted by their condition from taking a high level of exercise. However, they conclude that it seems likely that low levels of activity are an important factor in the development of obesity. As part of their efforts to tackle the obesity epidemic, governments should encourage physical activity, particularly of the more vigorous kind.

**Additional Information.:**

Please access these Web sites via the online version of this summary at http://dx.doi.org/10.1371/journal.pmed.0040097.

- The health risks of being overweight have been summed up by the Weight-Control Information Network
- A useful fact sheet on obesity is available from the World Health Organization
- The International Obesity Task Force is a network of organizations that seeks to alert the world to the growing health crisis threatened by soaring levels of obesity; the network's Web site has links to organizations involved in research on obesity or campaigning against it, plus useful publications
- MedlinePlus brings together authoritative information from the US National Library of Medicine, National Institutes of Health, and other government agencies and health-related organizations; there is a MedlinePlus page on obesity

## Introduction

The prevalence of childhood obesity is increasing in the United Kingdom [1], as it is across Europe [2], and in the United States [3]. This increase has important immediate and long-term health implications [4,5].

Obesity is fundamentally a result of chronic energy imbalance [6,7]. Diet survey data suggest that population levels of obesity have increased in the face of declining energy intake, implying that inactivity may be important in explaining the temporal trends in obesity [6,8]. While studies such as the National Heart Lung and Blood Institute's Growth and Health Study have reported associations between physical activity and obesity [9], the results of studies of the association between physical activity and obesity in children have been inconsistent [10]. This may reflect the fact that most studies have relied on inaccurate measures of physical activity or inaccurate measures of fat mass or both.

Physical activity in children is sporadic [11,12], and children are less able than adults to recall or record their physical activity, consequently questionnaires provide a poor measure of physical activity in children. In contrast objective techniques such as heart rate monitors or accelerometers have been shown to provide an accurate measure of physical activity in children [13,14]. Body mass index (BMI) is a measure of weight for height and is widely used to assess population levels of childhood obesity because it is easy to measure and because population standards are available for comparison. It does not, however, distinguish well between fat and lean mass across the normal range [15] unlike methods such as dual energy x-ray absorptiometry (DXA), which produce an estimate of lean mass, fat mass, and regional distribution of body fat [16].

We examined the association between physical activity (measured objectively using accelerometers), and fat and lean mass (measured using total body DXA), and BMI in a large population of contemporary children.

## Methods

### Study Population

The Avon Longitudinal Study of Parents and Children (ALSPAC) is a prospective study that has been described in detail elsewhere [17] (http://www.alspac.bris.ac.uk). Briefly, 14,541 pregnant women living in one of three Bristol-based health districts in the former County of Avon with an expected delivery date between April 1991 and December 1992 were enrolled in the study. Detailed information has been collected using self-administered questionnaires, data extraction from medical notes, and linkage to routine information systems and at research clinics. Ethical approval for the study was obtained from the ALSPAC Law and Ethics Committee and Local Research Ethics Committees.

### Measurement of Physical Activity

All children who attended the 11-year clinic were asked to wear an MTI Actigraph AM7164 2.2 accelerometer (Actigraph, http://www.theactigraph.com) for seven days. The Actigraph is an electronic motion sensor comprising a single plane (vertical) accelerometer. The Actigraph is small and light and is worn around the waist. Movement in a vertical plane is detected as a combined function of movement frequency and intensity and recorded as counts. The Actigraph has been validated in both children and adolescents against indirect calorimetry [18] observational techniques [19] and energy expenditure measured by doubly labelled water [20] and shown to be accurate. Actigraphs were initialised for each child using an Actigraph Reader Interface Unit (RIU-41A) with RIU software (version 2.26B, MTI Health Services, http://www.mtifwb.com). Children were asked to wear the Actigraph during waking hours and only to take it off for showering, bathing, or any water sports. Children were asked to record the times when they wore the Actigraph and time spent each day swimming or cycling, as the children did not wear the Actigraph when swimming, and the physical activity of cycling is not accurately recorded by the Actigraph. Actigraphs were returned by post and downloaded onto a PC using the Actigraph Reader Interface Unit and software.

### Measurement of Body Composition

Body composition was measured at the 11-year clinic. Height was measured with shoes and socks removed using a Harpenden stadiometer (Holtain, http://www.fullbore.co.uk/holtain/medical/welcome.html). Weight was measured using a Tanita TBF 305 body fat analyser and weighing scales (Tanita, http://www.tanita.co.uk). BMI was calculated as weight (in kilograms) divided by height squared (in metres). Fat mass and lean mass were measured using a Lunar Prodigy DXA scanner (GE Medical Systems, http://www.gehealthcare.com). Trunk fat mass was estimated using the automatic region of interest that included chest, abdomen, and pelvis. The scans were visually inspected and realigned where necessary.

### Potential Confounders

Age was the age the child attended the 11-year clinic. The 32-week antenatal questionnaire asked the mother to record her highest education level, which was categorised into none/Certificate of Secondary Education (CSE) (national school exams at age 16), vocational, O level (national school exams at age 16, higher than CSE), A level (national school exams at age 18), or degree. She also recorded the occupation of both herself and her partner, which were used to allocate them to social-class groups (classes I to V with III split into nonmanual and manual) using the 1991 Office for Population Censuses and Surveys classification; the lowest class of the mother and her partner was used in analysis. At enrolment, the mother was asked to record her height and prepregnancy weight, which were used to calculate the mother's BMI. The date of the last menstrual period as reported by the mother at enrolment and the actual date of delivery were used to estimate gestation. Infant sex and birthweight were recorded in the delivery room and abstracted from obstetric records and/or birth notifications. In the 18-week antenatal questionnaire, the mother was asked if she smoked in the first three months of pregnancy and in the last two weeks. In the 32-week antenatal questionnaire, the mother was asked how much she was currently smoking. Responses from the three trimesters were combined to create a variable for any smoking during pregnancy. In the 30-month questionnaire, the mother was asked how much time their child spent asleep at night (grouped into <10.5 or ≥10.5 hours), and in the 38-month questionnaire she was asked how much time they spent watching TV per week (grouped into ≤8 h or >8 h). A puberty questionnaire was filled in by the child's carer (usually the child's mother) when the child was approximately 11 years old, which included questions on pubertal stage [21]. Pubertal stage for boys was based on pubic hair development, and for girls was based on the most advanced stage for pubic hair and breast development.

### Measures of Physical Activity

Data from children who had worn the Actigraph for at least ten hours per day for at least three days were included. Two physical activity variables were used—total physical activity and time spent in moderate and vigorous physical activity (MVPA). Total physical activity was the total volume of physical activity and included activities at different intensities. Total physical activity was measured as the average counts per minute (cpm) over the period of valid recording. Total physical activity was used because this is the summary measure of total physical activity that has been validated against doubly labelled water [20]. MVPA was the average minutes of MVPA per valid day. Minutes of MVPA were used as current physical activity recommendations for children are framed in terms of time spent each day in MVPA [22]. We used a cut point of Actigraph output of greater than 3,600 cpm to define MVPA derived from a calibration study conducted in a subsample of 246 children who were asked to perform a series of everyday activities while wearing an Actigraph and a portable metabolic unit (Cosmed K4b^2^, Cosmed, http://www.cosmed.it). This estimate corresponded to four times resting metabolic rate that was achieved when children were walking briskly. This cut point was similar to that suggested recently in a study comparing different cut points [23]. Associations with total physical activity were calculated per 100 cpm as this difference is of a similar order to the differences observed between boys and girls. The associations with MVPA were calculated per 15 minutes of MVPA, as current recommendations are that children spend 60 minutes a day in MVPA [22]. Quintiles of MVPA and total activity were also used to look for a dose response by fitting the quintiles in a continuous model.

### Statistical Methods

Means and standard deviations (SDs) were calculated for continuous variables, and proportions were calculated for categorical variables. We used t-tests and Chi^2^ tests to compare differences between continuous and categorical values between children who provided physical activity data and those who did not. As MVPA, BMI, trunk fat, and fat mass had skewed distributions the median and interquartile range were calculated as summary measures, and logged BMI, trunk fat, and fat mass were used for calculation of the SD scores. Further analysis using continuous variables was based on internally derived SD scores (which are the same as Z-scores) for BMI, fat mass, lean mass, and trunk fat to allow comparison of the regression coefficients across outcome measures. Those in the top decile for fat mass after adjustment for age, height, and height squared were defined as obese. The cut points for the top decile of fat mass (for fat mass that has then been adjusted for age, height, and height squared for the sexes separately) was 17.9 kg in boys and 21.0 kg in girls. The associations with total physical activity and MVPA and the effects of potential confounding factors on the offspring outcomes were assessed using linear regression for continuous outcome variables and logistic regression for obesity. All associations except those with BMI were adjusted for height and height squared to take account of differences in stature (there was evidence of quadratic relationships with height). Previous studies have suggested that the association between physical activity and obesity is different in men and women [24,25]. We therefore formally tested the association between total physical activity and fat mass for an interaction with gender. As there was evidence of interaction (*p* = 0.005), we have carried out all analyses in boys and girls separately, and quintiles were derived separately for boys and girls. All analyses were performed using Stata version 8 (StataCorp, http://www.stata.com).

### Data Analysis Strategy

We selected possible confounding factors that were available on the whole cohort that have been shown to be independently associated with obesity in previous analyses [26,27]. We used a series of models to explore the possible role of confounders. In model 1 (minimally adjusted) we adjusted for age, height, and height squared (except for BMI) to take account of differences in age and height. In model 2 we adjusted for variables in model 1 plus confounding factors, i.e., factors that might be related to physical activity and obesity or that might be more distal determinants of physical activity—maternal education, social class, birthweight, gestational age, smoking in pregnancy, and obesity of mother in pregnancy. In model 3 we adjusted for the variables in model 2 plus factors that might be more proximal determinants of physical activity or might be proxy indicators of confounding factors – sleep pattern and TV viewing. In model 4 we adjusted for the variables in model 3 and the possible confounding effect of pubertal stage in those children with self-reported pubertal stage available within 16 weeks of their clinical assessment. We repeated the analyses in children who did not report swimming in the week of measurement and in children who did not report cycling in the week of measurement. We used the intraclass correlation coefficient derived from a repeat measures study in a subset of 315 children who wore the Actigraph on up to three subsequent occasions over the course of a year to take account of variation in usual physical activity and to adjust estimates for the effect of regression dilution bias [28]. We used Spearman correlation coefficients to describe the association between MVPA and total activity and fitted both of these variables together in unadjusted and adjusted models to try and examine the independent association of these two measures of activity.

## Results

A total of 11,952 children were invited to attend the research 11-year clinic. Of these, 7,159 (59.9%) came to the clinic, and 6,622 (92.5%) agreed to wear an Actigraph. Of the children who agreed to participate, 5,595 (84.5%) returned Actigraphs that satisfied the validity criteria. Estimates of body composition from the DXA scan were available on 5,500 children with valid physical activity measures.

The average age of the children seen in the 11-year clinic was 141 months, so we have referred to them as 12-year-old children. The characteristics of these children are summarised in Tables 1 and 2. Objectively measured physical activity levels were higher in boys than girls, 663 versus 605 cpm (*p* < 0.001). The children who attended the clinic were more likely to be girls, have a higher birthweight, be from a higher social class, have older and taller mothers, and have mothers with higher levels of education (unpublished data). There was a similar pattern of differences between those who provided valid measures of physical activity and those who attended the clinic but did not provide valid physical activity data (unpublished data). Pubertal stage was associated with lower levels of physical activity, higher lean mass, and higher odds of obesity in girls and with higher lean mass in boys (unpublished data).

**Table 1:**
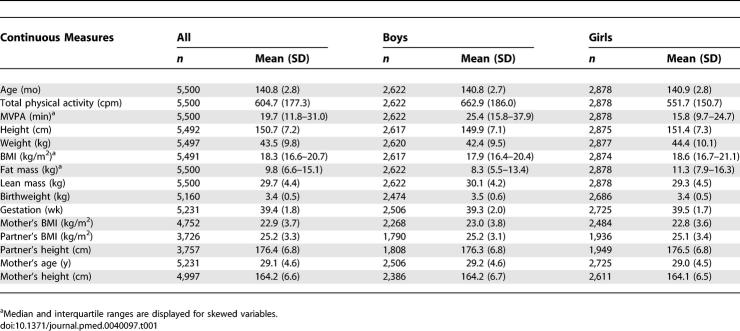
Continuous Measures in the 5,500 12-Year-Old Children with Objective Measures of Physical Activity and DXA Measures of Body Composition from ALSPAC

**Table 2:**
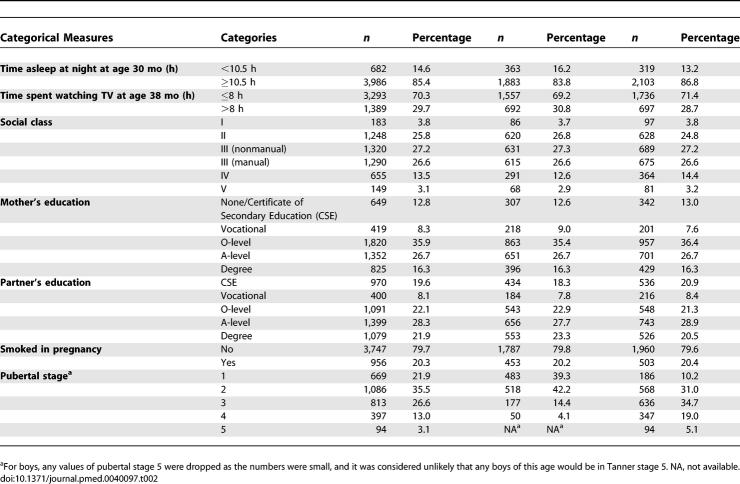
Categorical Measures in the 5,500 12-Year-Old Children with Objective Measures of Physical Activity and DXA Measures of Body Composition ALSPAC

The associations between measures of body composition and total physical activity measured as a difference of 100 cpm are shown in Table 3. BMI, fat mass, and trunk fat were negatively associated with total physical activity, and lean mass was positively associated with total physical activity. The association with fat mass was not altered by adjustment for confounding factors. The associations with BMI showed a similar pattern. The association between physical activity and lean mass was not affected by adjustment for confounding factors. The intraclass correlation coefficient estimated in a calibration study conducted in this population that measured physical activity in a subset of study children on three further occasions was 0.53 in both sexes combined. When this was used to correct the observed association between physical activity and fat mass for measurement error, the regression coefficient of the SD score for fat mass per 100 cpm increased from −0.11 to −0.22 in model 1 in boys and from −0.08 to −0.15 in model 1 in girls.

**Table 3:**
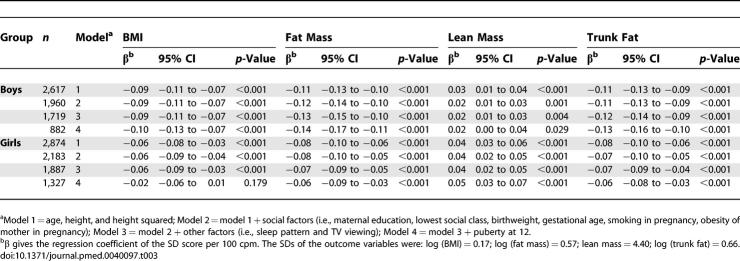
The Association between Objectively Measured Total Physical Activity and Body Composition SD Scores in 5,500 11-Year-Old Children from ALSPAC

The association between measures of body composition and a difference of 15 minutes of MVPA is shown in Table 4. The results were similar to those observed for total physical activity. The intraclass correlation coefficient was 0.45 in both sexes combined, lower than that for total physical activity. When this was used to correct the observed association between physical activity and fat mass for measurement error, the regression coefficient of the SD score for fat mass per 15 minutes of moderate or vigorous physical activity increased from −0.23 to −0.52 in model 1 in boys and from −0.17 to −0.39 in model 1 in girls.

**Table 4:**
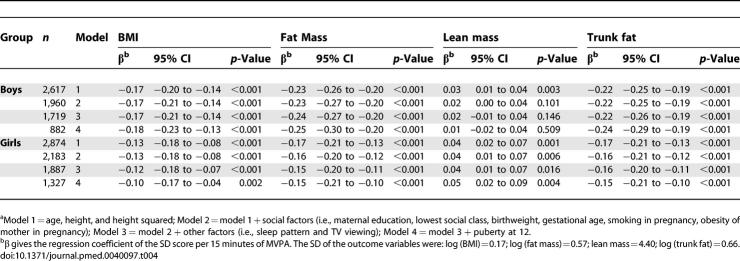
The Association between Objectively Measured Minutes of MVPA and Body Composition SD Scores in 5,500 12-Year-Old Children from ALSPAC

To explore the independent associations with fat mass of total activity and MVPA, we fitted both variables to the same models. MVPA and total physical activity were highly correlated, with coefficients of 0.87, *p* < 0.0001 in boys and 0.83, *p* < 0.0001 in girls. When we fitted the two variables together, the standard error (SE) doubled for both MVPA and total activity. The association with total activity disappeared completely, while that with MVPA remained unaltered. In model 1 in boys, the regression coefficients ± SEs were −0.11 ± 0.010 and −0.23 ± 0.020 for total activity and MVPA, respectively when fitted separately, and were 0.03 ± 0.016 and −0.28 ± 0.032 when both were fitted in the same model. The effect of combined adjustment was similar in girls and in fully adjusted models in boys and girls (unpublished data). We therefore present the rest of the results (including the analyses run on various different groups and with further adjustment for lean mass) for MVPA and obesity rather than total physical activity and obesity

**Figure 1:**
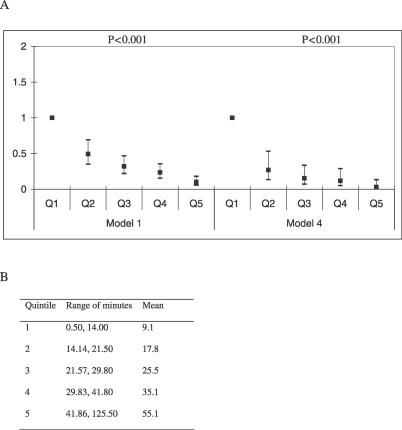
Adjusted Odds Ratios for Obesity by Quintiles of Objectively Measured Minutes of Moderate and Vigorous Activity in 2,622 12-Year-Old Boys from ALSPAC (A) Model 1 gives the odds ratios from the minimally adjusted model, i.e., obesity (adjusted for age, height, and height squared) regressed on quintiles of MVPA. Model 4 gives the odds ratios from the maximally adjusted model, i.e., obesity (adjusted for age, height, and height squared) regressed on quintiles of MVPA, maternal education, lowest social class, birthweight, gestational age, smoking in pregnancy, obesity of mother, sleep pattern, TV viewing, and pubertal stage. (B) Mean and ranges of quintiles of minutes of moderate and vigorous activity for boys.

**Figure 2:**
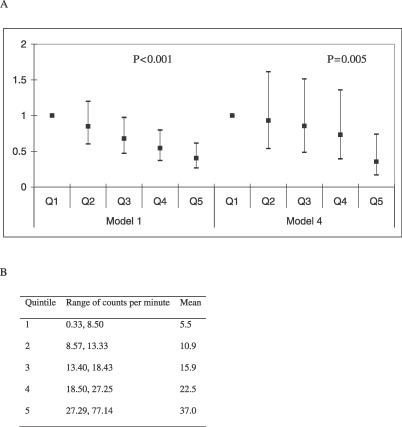
Adjusted Odds Ratios for Obesity by Quintiles of Objectively Measured Minutes of Moderate and Vigorous Activity in 2,878 12-Year-Old Girls from ALSPAC (A) Model 1 gives the odds ratios from the minimally adjusted model, i.e., obesity (adjusted for age, height, and height squared) regressed on quintiles of MVPA. Model 4 gives the odds ratios from the maximally adjusted model, i.e., obesity (adjusted for age, height, and height squared) regressed on quintiles of MVPA, maternal education, lowest social class, birthweight, gestational age, smoking in pregnancy, obesity of mother, sleep pattern, TV viewing, and pubertal stage. (B) Mean and ranges of quintiles of minutes of moderate and vigorous activity for girls

The odds ratio for obesity in boys for a difference of 15 minutes of MVPA was 0.45 (95% confidence interval [CI] 0.38–0.53, *p* < 0.0001) in model 1 and 0.30 (95% CI 0.20–0.43, *p* < 0.0001) in model 4. The odds ratio for obesity in girls for a difference of 15 minutes of MVPA was 0.62 (95% CI 0.52–0.75, *p* < 0.0001) in model 1 and 0.61 (95% CI 0.45–0.83, *p* = 0.002) in model 4. The odds ratio of obesity for quintiles of minutes of MVPA in boys is shown in Figure 1, and the odds ratio of obesity for quintiles of minutes of MVPA in girls is shown in Figure 2. In model 1 the odds ratio for obesity between top and the bottom quintiles of minutes of MVPA was 0.10 (95% CI 0.06–0.18, *p* for trend <0.0001) in boys and 0.40 (95% CI 0.27–0.61, *p* for trend <0.0001) in girls. In model 4 the odds ratio for obesity between top and the bottom quintile of MVPA was 0.03 (95% CI 0.01–0.12, *p* for trend <0.0001) in boys and 0.36 (95% CI 0.17–0.74, *p* for trend = 0.006) in girls.

We repeated the analyses in 1,742 (71%) of the boys and 1,715 (64%) of the girls who had not reported swimming in the week physical activity was recorded, and the results were similar to those presented above (unpublished data). We repeated the analyses in 1,719 (70%) of the boys and 2,081 (78%) of the girls who had not reported cycling in the week physical activity was recorded, and the results were similar to those presented above (unpublished data). We reran model 1 in those with complete data on all confounders, and the regression coefficients were essentially the same as those presented above (unpublished data). We reran model 4 adjusting for lean mass, and the results were not altered (unpublished data).

## Discussion

We found a strong negative dose-response association between objectively measured physical activity and DXA-derived measures of fat mass and obesity. The associations between physical activity and trunk fat were very similar to those observed between physical activity and fat mass, which is not surprising as trunk mass and fat mass were very closely correlated in this population. We also found a strong negative association between physical activity and BMI that was weaker than that observed for fat mass because of a positive association between physical activity and lean mass. The association between physical activity and fat mass was stronger for boys than girls. The associations between MVPA and fat mass were unaltered after adjustment for total physical activity, but the associations between total physical activity and fat mass disappeared when we adjusted for MVPA. These associations are unlikely to be due to chance as the *p*-values were fairly small, and the confidence intervals were narrow.

Two prospective studies have examined the association between objectively measured activity and obesity [29,30]. The first measured 103 children from the United States aged three to five years for three to five days with a Caltrac movement sensor (http://www.muscledynamics.net) annually for eight years and found that the final sum of skin folds was associated with activity over the follow-up period [29]. The second of these measured 454 American Indian children (average age 7.5 years) using a tri-axial movement sensor on one day and followed them up three years later [30]. There was an association between total activity and obesity in children who were normal weight at baseline but not in those who were overweight. This association was stronger for a measure of body fat derived from skin folds and bio-impedance than for BMI [30]. Though both of these studies reported a protective association between physical activity and obesity, neither was large enough to examine whether there was dose-response association or to compare the associations between boys and girls or between moderate and total physical activity.

The association between objectively measured physical activity and measures of obesity based on BMI and skin fold measurements was examined in two large cross-sectional studies (*n* > 1,000) [31,32]. In the first study, 1,292 children, aged nine to ten years, were studied from four distinct regions in Europe (Denmark, Portugal, Norway, and Estonia). Physical activity was measured using the Actigraph with a similar protocol to that employed in our study. There were associations between total physical activity and time spent in MVPA in vigorous activity and obesity, but these associations were considerably weaker than the associations we observed in our population [31]. In the second study, 1,553 ten- to 14-year-old girls from the United States were studied. Physical activity was measured using the Actigraph worn for six days, and the obesity was measured using BMI and triceps skinfold thickness. There were associations between percentage body fat and minutes of MVPA [32]. Both of these studies showed a negative association between physical activity and obesity, but the associations were weaker than those we observed. The measures of physical activity were similar, and the cut points for vigorous physical activity used in the European study and used for MVPA in the United States-based study were similar to those we used for MVPA. Though the associations may vary across populations and at different ages, we think that the fact that we found stronger associations for fat mass than BMI suggests that the accuracy of the measure of obesity used may in part explain the observed differences.

Only one study has used an objective measure of physical activity and an accurate measure of obesity [33]. In this study 248 Swedish school children aged eight to 11 wore Actigraphs for up to four days, and percentage body fat was measured using DXA. The odds of obesity (defined as one SD above the mean percentage body fat) in the least activity quartile was 4.0 (95% CI 1.2–13.5). The association with obesity was stronger with vigorous activity (defined as >3,498 cpm) than moderate activity (defined as >1,670 cpm and <3,498 cpm). Our results are thus consistent with these, suggesting that there is a strong cross-sectional association between physical activity and obesity, and that it is stronger for higher intensity physical activity.

If causal, the associations we have demonstrated are of potential public health importance. Our data suggest that a modest increase in physical activity of 15 minutes of MVPA is associated with lower odds of obesity of over 50% in boys and nearly 40% in girls. Though total physical activity and MVPA were closely correlated, suggesting that children with high levels of MVPA have high levels of total physical activity, our data provide empirical support for the current physical activity recommendations for children that are framed in terms of MVPA rather than total physical activity [22].

Our finding that the association between physical activity and obesity was stronger in boys than girls was a prespecified analysis based on findings from studies in adults [24,25,34]. We are not aware of any previous reports in children. Our results suggest that though higher levels of physical activity are associated with reduced risk of obesity in both boys and girls, the strength of the association between physical activity level and obesity differs between boys and girls. This may be because physical activity has a stronger effect on appetite and satiety in boys, or it may be that girls use dietary restraint more than boys to regulate their weight.

Our study has a number of limitations. First, our study is cross-sectional and we cannot therefore rule out the possibility that these associations represent reverse causality, and that obesity leads to a reduction in physical activity. The fact that these associations were observed across the range of fat mass rather than just in obese children makes this explanation less likely. Even if the associations are due to reverse causality and obesity leads to reduced activity, this is itself an important observation as reduced physical activity in obese people may increase the morbidity and mortality associated with obesity. Second, these data are observational, and it is possible that confounding could explain our results. Though the observed associations could be due to confounding we think this is unlikely as physical activity in this cohort is weakly negatively associated with higher social position (unpublished data), and the associations were largely unaltered by adjustment for a number of confounding factors. More recent measures of possible confounders such as social position were not available, and these could explain these associations. Third, these data are based on a single measure of activity over a three- to seven-day period that didn't necessarily include a weekend day. Though some studies have used longer reporting periods, many studies have included children with three days or fewer [29,30,31,33], and the association between physical activity and obesity was similar in children with different numbers of days of valid recording (unpublished data). Shorter recording periods will measure usual physical activity less accurately and therefore attenuate physical activity–obesity associations; we have used the intraclass correlation coefficient based on repeat measures over the course of a year to quantify the likely effect of such measurement error. Fourth, we used one-minute epochs to define activity level, and we may therefore have underestimated the total amount of MVPA where this is sporadic rather than sustained. It is reassuring, therefore, that our results were similar to those reported in a study using ten-second epochs [33]. Finally, we were not able to collect data on physical activity or body composition on a substantial number of children originally enrolled in the study. These missing data will result in reduced power, which is not a particular problem in a study of this size. Potentially more importantly, missing data can lead to bias if the association between physical activity and obesity is different in the children who did not take part. While we cannot exclude bias due to missing data, the fact that the associations were not altered by adjustment for factors associated with missing data provides some reassurance. Further, although attendance at the 11-year clinic was associated with markers of higher social position, physical activity showed a weak negative association with social position (unpublished data).

In conclusion we have shown a strong negative dose-response association between objectively measured physical activity and childhood obesity measured as fat mass and BMI. Our findings, if confirmed, suggest that public health policies that increase physical activity levels and in particular MVPA in children may help to reduce the prevalence of childhood obesity. These associations suggest even a modest increase of 15 minutes MVPA might result in an important reduction in the prevalence of overweight and obesity. Prospective studies are required to confirm these associations and to describe how physical activity-obesity associations vary over time.

## Abbreviations

ALSPAC: Avon Longitudinal Study of Parents and Children
BMI: body mass index
CI: confidence interval
cpm: counts per minute
DXA: dual energy x-ray absorptiometry
MVPA: moderate and vigorous physical activity
SD: standard deviation
SE: standard error
